# A Case of Lobar Pneumonia and Its Treatment

**Published:** 1899-05-13

**Authors:** G. A. Sutherland

**Affiliations:** Physician to Paddington Green Children's Hospital


### Hospital Clinics and Medical Progress.

### A CASE OF LOBAR PNEUMONIA AND ITS TREATMENT.

By G. A. Sutherland, M.D., M.R.C.P., Physician to Paddington Green Children's Hospital.

Recently there has been a very instructive case under my care which has presented a considerable number of the possible deviations from the normal course.

The case is that of a boy 18 months old, who was admitted on December 27th, 1898, suffering from pneumonia. He was an extremely pale, rhachitic child, and the history of his diet, breast milk for 10 months and Nestlé's milk subsequently, was sufficient to explain the presence of rickets. The acute illness had developed suddenly five days before admission, when he became feverish and lost his appetite. The signs of pneumonia were well marked over the lower lobe of the right lung, where, from the inferior angle of the scapula there was marked dulness, with bronchial breathing and numerous fine crepitations. The temperature was 104 deg. F., and both the respirations and pulse rate were much increased in frequency. The diagnosis of right lobar pneumonia was therefore clear, the only complication present being cardiac weakness. During the next 11 days we were able to trace the gradual extension of the pulmonary consolidation upwards until the whole of the right lung was solidified, but before the extreme apex was affected signs of resolution were present at the base. The last part to be affected was the middle lobe. Although there were no symptoms suggestive of middle ear trouble, I followed our usual custom in cases of pneumonia, and asked Dr. Lambert Lack to examine the ears. This he did on the eighth day after admission, and found both membranes bulging with evidence of pus in the right middle ear. He therefore punctured both membranes, some thick pus escaping from the right, and ordered the ears to be syringed with warm boracic lotion. On the following day there was a fall in the temperature to 100 deg., but as it rose again the same night it was evidently not the ear trouble which was maintaining the pyrexia. Two days later—*i.e.*, 11 days after admission, or 16 days after the outset, there was a real crisis, and a marked improvement in the child's general condition. The previously apathetic patient, who lay occupied solely with his breathing, now sat up in bed and took an interest in what was going on around. There was a slight discharge from both ears for a few days, but this passed off under the syringing.

This, however, did not represent the end of the illness. A few days later the temperature rose again, although not to the same height as before, and signs of pneumonia were detected at the left apex, which gradually spread until the whole of the upper lobe of the left lung was consolidated. During this attack the child did not present the same evidences of prostration as before, but the anæmia was progressive. The resolution of the pneumonia in the right lung was proceeding satisfactorily, with the exception of the middle lobe, where the dulness and tubular breathing were very persistent. In due course signs of resolving appeared in the left upper lobe, and everything seemed to be going on satisfactorily when suddenly the temperature rose again on the 39th day of the illness, and commencing consolidation was detected in the lower lobe of the left lung. As the pneumonia had invaded in succession every part, first of the right, and then of the left lung, we now hoped that if the child could be tided over this last attack a favourable issue might be expected, the general condition being satisfactory considering the prolonged and severe nature of the illness. Unfortunately, however, the pneumonia in the lower lobe of the left lung did not clear up in the normal way, and Mr. Goulden, the house physician, finding at the left base marked dulness with distant breath sounds, very properly explored that region with a syringe suspecting the presence of fluid, possibly pus—a possibility always to be remembered in cases of lobar pneumonia where the ordinary signs of resolution do not appear soon after the crisis. No fluid was obtained on this occasion, and the cause of this failure will be explained later. The child continued in much the same condition until on the morning of the 47th day of the illness he had a convulsive seizure with vomiting and unconsciousness, followed an hour later by another. The left side of the body was affected in these convulsions, face, arm, and leg, with conjugate deviation of the eyes to the left. When I saw him the same evening he was quite conscious, and had had no return of the convulsions or vomiting. The left arm and leg were paretic, but not paralysed. The breathing was rapid, rather laboured, and of an irregular type, suggesting cerebral disturbance. The cardiac action was good. Over the left lower lobe the percussion note was very flat, and the breath sounds very feeble. There were no signs of the presence of a large quantity of fluid, such as displacement of neighbouring organs, &c., but still suspecting the presence of pus, I explored the left base in two places; again with a negative result. The child was evidently sinking, and died on the following day from respiratory failure, without developing any further acute symptoms.

I wish to direct attention specially to the conditions found at the necropsy, because in them we have definite proof of the lesions diagnosed during life, and from them we gather information as to some points which were not ascertained during life. The right lung had cleared up entirely with the exception of the middle lobe, which was in a state of grey hepatisation. The left lung was surrounded by soft pleural adhesions which separated easily, but over the lower lobe posteriorly, and strictly limited superiorly by the upper margin of that lobe there was a layer of purulent semi-solid exudation about half an inch thick, which, when the lung was removed, separated into two layers, one adherent to the visceral and the other to the parietal pleura. There was no fluid present, and it was thus explained how the exploration of this region during life had failed, as the needle had passed through the layer of pus into the lung beneath. The left lower lobe was in a state of red hepatisation, while the upper lobe was practically healthy. On removing the skull cap and dura mater the surface of the brain was found to be covered with a thin layer of pus lying underneath the arachnoid. This pus was most abundant around the longitudinal fissure and the large sulci; but the anterior two-thirds of the brain, both superiorly and inferiorly, were covered with pus. The surface of the brain was intensely congested, and the cerebral substance was softened and œdematous. On opening the right ear there was no evidence of any disease, but the left middle ear and surrounding mastoid cells were filled with pus similar to that lining the surface of the brain.

(*To be continued.*)

